# Supplementary material for: Effects of Additional Granola in Children’s Breakfast on Nutritional Balance, Sleep and Defecation: An Open-Label Randomized Cross-Over Trial
Source: Children (Basel). 2023 Apr 25;10(5):779. doi: 10.3390/children10050779 (PMC10217378; doi:10.3390/children10050779)
Supplement: Supplementary file 1 [file children-10-00779-s001.zip › children-2318682-supplementary.pdf]

Supplemental data

Table S1. Characteristic of subjects in each group

	Group A	Group B	
Age (years)	10.1 ± 1.4	10.4 ± 1.6	*p=0.8790*
Height (cm)	138.4 ± 14.0	139.3 ± 12.2	*p=0.8515*
Weight (kg)	34.1 ± 12.7	35.0 ± 9.4	*p=0.5800*

Figure S1. Appearance of granola snack (26 g)

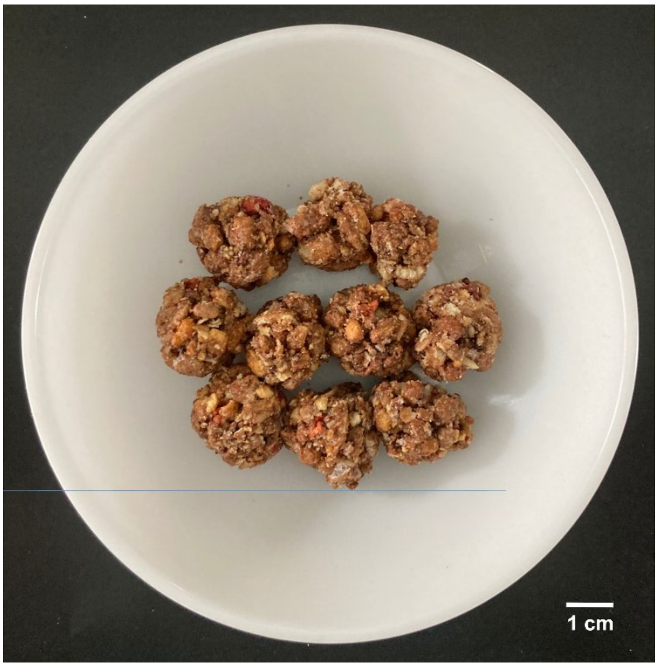

Table S2. Nutrition of test food

**Macronutrients**	
Amount	26.0 g
Energy	119.6 kcal
Protein	4.1 g
Fat	5.5 g
Cholesterol	0.0 g
Carbohydrate	14.9 g
Sugar	11.8 g
Dietary Fiber	3.1 g
Salt Equivalent	0.1 g
**Minerals & Vitamins**	
Potassium	203.0 mg
Calcium	50.0 mg
Phosphorus	69.0 mg
Iron	2.4 mg
Vitamin A	118.0 μg
Vitamin D	0.8 μg
Vitamin B_1	0.2 mg
Niacin	2.5 mg
Vitamin B_6	0.2 mg
Vitamin B_{12}	0.4 μg
Folate	44.0 μg
Pantothenic Acid	0.7 mg

Table S3A. Nutritional adequacy of breakfast in control and intervention periods

Macronutrients	Control	+Granola		*p value*
Energy	69.6 ± 4.0	84.1 ± 4.0	****	*0.0002*
Protein	83.6 ± 4.7	93.9 ± 2.3	**	*0.0020*
Total Fat	85.6 ± 4.6	99.9 ± 3.0	**	*0.0027*
Carbohydrate	72.2 ± 4.3	81.2 ± 3.7	*	*0.0289*
SFA	104.7 ± 2.8	105.0 ± 2.1		*0.6875*
n-6 PUFA	58.0 ± 4.6	52.4 ± 4.3		*0.1790*
n-3 PUFA	58.5 ± 6.4	51.1 ± 5.7		*0.1815*
Dietary Fiber	72.0 ± 5.9	98.4 ± 0.9	***	*0.0001*
**Minerals & Vitamins**				
Salt Equivalent	120.5 ± 11.8	108.8 ± 4.2		*0.5781*
Potassium	80.1 ± 5.0	93.0 ± 2.8	**	*0.0010*
Calcium	64.0 ± 6.0	82.8 ± 5.0	****	*<0.0001*
Magnesium	65.4 ± 5.9	62.3 ± 5.6		*0.6507*
Phosphorus	70.2 ± 5.5	82.9 ± 4.6	***	*0.0002*
Iron	56.2 ± 5.9	94.7 ± 2.7	****	*<0.0001*
Zinc	74.2 ± 5.9	72.2 ± 5.2		*0.8052*
Copper	79.2 ± 5.6	77.9 ± 5.5		*0.6788*
Manganese	54.1 ± 6.2	47.5 ± 6.1		*0.0647*
Vitamin A (Retinol)	68.8 ± 10.9	68.8 ± 6.5		*0.3737*
Vitamin D	61.2 ± 7.0	87.6 ± 3.9		*0.0012*
Vitamin E-α	81.8 ± 5.4	80.3 ± 5.7		*0.7869*
Vitamin K	62.5 ± 8.2	57.2 ± 7.9		*0.4873*
Vitamin B_1	60.9 ± 4.8	93.6 ± 2.4	****	*<0.0001*
Vitamin B_2	71.5 ± 6.3	71.3 ± 6.0		*0.7032*
Niacin	93.6 ± 3.3	95.9 ± 2.5		*0.1875*
Vitamin B_6	71.8 ± 5.9	94.4 ± 2.6	***	*0.0001*
Vitamin B_{12}	83.0 ± 6.3	95.4 ± 2.8	*	*0.0156*
Folate	83.2 ± 5.5	100.3 ± 1.1	**	*0.0022*
Pantothenic Acid	68.2 ± 5.6	89.1 ± 3.3	****	*<0.0001*
Vitamin C	58.7 ± 7.4	70.9 ± 7.1	**	*0.0067*

Mean ± SEM, *: $p<0.05$, **: $p<0.01$, ***: $p<0.001$, ****: $p<0.0001$.

Table S3B. Nutritional intake of breakfast in control and intervention periods

**Macronutrients**	Control	+Granola
Energy (kcal)	387.4 ± 19.9	486.5 ± 28.4
Protein (g)	13.3 ± 1.2	17.1 ± 1.3
Total Fat (g)	13.2 ± 1.0	18.2 ± 1.1
Carbohydrate (g)	54.8 ± 3.2	64.6 ± 4.0
SFA (g)	4.7 ± 0.5	4.6 ± 0.6
n-6 PUFA (g)	1.5 ± 0.1	1.4 ± 0.1
n-3 PUFA (g)	0.4 ± 0.1	0.3 ± 0.0
Dietary Fiber (g)	3.3 ± 0.4	6.9 ± 0.6
**Minerals & Vitamins**		
Salt Equivalent (g)	1.6 ± 0.3	1.5 ± 0.1
Potassium (mg)	521.6 ± 67.2	710.6 ± 59.9
Calcium (mg)	159.5 ± 23.3	221.2 ± 23.5
Magnesium (mg)	46.3 ± 7.2	39.7 ± 4.6
Phosphorus (mg)	239.6 ± 28.3	297.4 ± 27.7
Iron (mg)	1.5 ± 0.2	4.1 ± 0.4
Zinc (mg)	1.6 ± 0.2	1.4 ± 0.1
Copper (mg)	0.2 ± 0.0	0.2 ± 0.0
Manganese (mg)	0.5 ± 0.1	0.4 ± 0.1
Vitamin A (Retinol) (μg)	155.5 ± 48.9	150.5 ± 21.7
Vitamin D (μg)	1.9 ± 0.4	2.2 ± 0.2
Vitamin E-α (mg)	1.6 ± 0.1	1.5 ± 0.1
Vitamin K (μg)	36.7 ± 7.7	30.8 ± 6.5
Vitamin B_1 (mg)	0.2 ± 0.0	0.4 ± 0.0
Vitamin B_2 (mg)	0.3 ± 0.0	0.3 ± 0.0
Niacin (mg)	5.0 ± 0.5	5.8 ± 0.7
Vitamin B_6 (mg)	0.3 ± 0.0	0.5 ± 0.0
Vitamin B_{12} (μg)	1.1 ± 0.2	1.2 ± 0.1
Folate (μg)	69.4 ± 11.6	110.8 ± 9.3
Pantothenic Acid (mg)	1.4 ± 0.2	2.0 ± 0.2
Vitamin C (mg)	26.6 ± 7.9	21.3 ± 3.1

Mean ± SEM.

Table S4. Sleep parameters in control and intervention periods

		Control			+Granola		
**Sleep duration (min)**	Weekday	528.0	±	48.1	522.3	±	53.1 #
	Weekend	564.0	±	59.3 ††	551.0	±	54.3 #, ††
**Bedtime**	Weekday	22:20	±	54.7 min	22:15	±	56.9 min
	Weekend	22:27	±	51.4 min †	22:31	±	52.1 min †
**Wake up time**	Weekday	7:08	±	24.7 min	6:57	±	34.2 min ##
	Weekend	7:49	±	44.8 min †††	7:34	±	54.5 min ##, †††
**Sleep-corrected MSF**		2:57	±	32.5 min	2:55	±	40.2 min
**Social jet lag (min)**		24.2	±	19.4	26.2	±	34.5

Mean ± SD, #, ## Significant differences due to the intervention effect, $p<0.05$, $p<0.01$. †, ††, ††† Significant differences due to the day-of-week effect, $p<0.05$, $p<0.01$, $p<0.001$.

Table S5. Defecation status in control and intervention periods

		Control			+Granola				*p value*
**Defecation frequency (times/week)**		6.5	±	2.9	7.7	±	3.3	**	*0.004*
**Defecation frequency (days/week)**	7 days	12			12				*0.123*
	5-6 days	2			7				
	3-4 days	6			2				
	0-2 days	1			0				
**BSS score**		3.4	±	0.9	3.6	±	0.8		*0.250*
**CAS total score**		1.3	±	1.6	1.4	±	1.6		*0.862*

Mean ± SD, **: $p<0.01$.
